# The Composition and Cellular Sources of CSPGs in the Glial Scar After Spinal Cord Injury in the Lamprey

**DOI:** 10.3389/fnmol.2022.918871

**Published:** 2022-06-27

**Authors:** Guixin Zhang, Li-Qing Jin, William Rodemer, Jianli Hu, Zachary D. Root, Daniel M. Medeiros, Michael E. Selzer

**Affiliations:** ^1^Shriners Hospitals Pediatric Research Center (Center for Neural Repair and Rehabilitation), Department of Neural Sciences, Philadelphia, PA, United States; ^2^Department of Ecology and Evolutionary Biology, University of Colorado, Boulder, CO, United States; ^3^Department of Neurology, The Lewis Katz School of Medicine at Temple University, Philadelphia, PA, United States

**Keywords:** CSPGs, lecticans, lamprey, spinal cord injury, glial scar

## Abstract

Axon regrowth after spinal cord injury (SCI) is inhibited by several types of inhibitory extracellular molecules in the central nervous system (CNS), including chondroitin sulfate proteoglycans (CSPGs), which also are components of perineuronal nets (PNNs). The axons of lampreys regenerate following SCI, even though their spinal cords contain CSPGs, and their neurons are enwrapped by PNNs. Previously, we showed that by 2 weeks after spinal cord transection in the lamprey, expression of CSPGs increased in the lesion site, and thereafter, decreased to pre-injury levels by 10 weeks. Enzymatic digestion of CSPGs in the lesion site with chondroitinase ABC (ChABC) enhanced axonal regeneration after SCI and reduced retrograde neuronal death. *Lecticans* (aggrecan, versican, neurocan, and brevican) are the major *CSPG* family in the CNS. Previously, we cloned a cDNA fragment that lies in the most conserved link-domain of the lamprey lecticans and found that lectican mRNAs are expressed widely in lamprey glia and neurons. Because of the lack of strict one-to-one orthology with the jawed vertebrate lecticans, the four lamprey lecticans were named simply A, B, C, and D. Using probes that distinguish these four lecticans, we now show that they all are expressed in glia and neurons but at different levels. Expression levels are relatively high in embryonic and early larval stages, gradually decrease, and are upregulated again in adults. Reductions of lecticans B and D are greater than those of A and C. Levels of mRNAs for lecticans B and D increased dramatically after SCI. Lectican D remained upregulated for at least 10 weeks. Multiple cells, including glia, neurons, ependymal cells and microglia/macrophages, expressed lectican mRNAs in the peripheral zone and lesion center after SCI. Thus, as in mammals, lamprey lecticans may be involved in axon guidance and neuroplasticity early in development. Moreover, neurons, glia, ependymal cells, and microglia/macrophages, are responsible for the increase in CSPGs during the formation of the glial scar after SCI.

## Introduction

Chondroitin sulfate proteoglycans (CSPGs) are large glycoproteins that have been widely implicated in suppressing axon regrowth after injury to the central nervous system (CNS). They consist of a protein core and a chondroitin sulfate side chain containing repeating glucuronic acid and N-acetyl-galactosamine disaccharide polymers. CSPGs are widely expressed in the normal CNS, serving as guidance cues during development, and as major structural components of the perineuronal nets (PNNs) that are responsible for synaptic stabilization in the normal adult (Brittis et al., 1992; Celio and Blumcke, 1994; Celio et al., 1998). The CSPGs of the PNNs are the lecticans (Yamaguchi, 2000; Deepa et al., 2006), four hyaluronan-binding proteoglycans called “aggrecan” (ACAN), “brevican” (BCAN), “neurocan” (NCAN), and “versican” (VCAN; Ruoslahti, 1996; Oohira et al., 2000; Yamaguchi, 2000; Rauch, 2004). Lecticans have an Ig-like loop and two link modules at the amino terminus, an epidermal growth factor (EGF) C-type lectin domain (CLECT), also known as the “carbohydrate-recognition domain” (CRD), and a complement control protein domain (CCP) at the carboxy end. ACAN has two additional link modules. These lecticans differ mainly in the number of chondroitin sulfate chains in the central domain. Since their first description, the cellular origin of PNNs has been the subject of considerable debate (Celio et al., 1998). Immunohistochemical experiments with lectins and antibodies recognizing CSPGs at both light and electron microscopic levels reveal reaction products interposed between and on neuronal surfaces (Hockfield and McKay, 1983; Nakagawa et al., 1986; Zaremba et al., 1989; Bertolotto et al., 1991; Bruckner et al., 1993; Schweizer et al., 1993). However, because these reagents could bind extracellular epitopes present on molecules with transmembrane domains, immunoreactive constituents have been interpreted by various investigators as being localized to glial endfeet, the neuronal surface, or the extracellular space in between (Lafarga et al., 1984; Celio and Blumcke, 1994; Blumcke et al., 1995). The glial nature of this structure was widely accepted (Brauer et al., 1982, 1984; Celio and Blumcke, 1994; Viggiano et al., 2000).

Immunohistochemistry (IHC) with several CSPGs antibodies showed that different subsets of neurons express different types of cell surface CSPGs, suggesting that CSPGs may variably regulate the neuronal extracellular microenvironment (Lander et al., 1998), and also that neurons may contribute to their own extracellular matrix. However, because IHC may suffer from antibody cross-reactivity, antigens can be difficult to identify with certainty. And because the CSPGs in PNNs are cell-surface proteins, the results cannot distinguish unambiguously whether the proteins originate in neurons or adjacent glia. Moreover, since the antibodies commonly used are not specific for any subset of CSPGs, the type of CSPGs that derive from neurons are not known. The use of *in situ* hybridization (ISH) can avoid these ambiguities. When expressions of PNNs were examined in rat cerebellum by both IHC and ISH, it was found that NCAN and ACAN mRNAs are expressed in neurons of the cerebellar cortex that have PNNs (Carulli et al., 2006). Thus, neurons may contribute to the formation of PNNs.

In mammals, CSPGs also contribute to the formation of the glial scar that acts as a barrier against new axon growth into the injury site (McKeon et al., 1991; Properzi et al., 2003; Galtrey and Fawcett, 2007; Siebert et al., 2014). Enzymatic degradation of CSPGs with chondroitinase ABC (ChABC) after spinal cord injury (SCI) enhances axonal growth and functional recovery (Bradbury et al., 2002; Warren et al., 2018a). Immunoreactivities for NCAN, BCAN, and VCAN are increased in the SC parenchyma surrounding a lesion within days, peak at 2 weeks (weeks), and persist at least 4 weeks post-injury (Jones et al., 2003). The cellular sources of lecticans that contribute to the glial scar are believed to be activated astrocytes (Fidler et al., 1999; Powell and Geller, 1999). Whether neurons surrounding or in the transection (TX) site secrete lecticans is not clear.

We had previously searched the lamprey whole-genome sequencing (Trace Archive Nucleotide for Petromyzon marinus-WGS) and Ensembl genome databases, using the basic local alignment search tool (BLAST) on the server of the National Center for Biotechnology Information (NCBI), found several lectican-like contigs, none of which were clearly homologous with any of the known members of the lectican family. Searching the sea lamprey germline genome (Smith et al., 2018b) and their own sequencing data, others recently published four sequences similar to those of jawed vertebrate lecticans (Root et al., 2021). Because the sequences lacked strict one-to-one orthology with mammalian lecticans, the authors named the lamprey lecticans A, B, C, and D. After a recent re-assembly of the lamprey genome (Feb., 2020), several lecticans of sea lamprey were predicted by automated computational analysis and were annotated and made available in NCBI databases. To clarify the relationship between the newly annotated lecticans and the four assembled sequences, in the present article we have performed an alignment and phylogenetic analysis.

We have found several CSPG receptors in the lamprey genomic database (Zhang et al., 2014a; Rodemer et al., 2020), and previously cloned the full-length of two receptor protein tyrosine phosphatases (RPTPs) protein tyrosine phosphatase sigma (PTPσ) and leukocyte antigen-related protein tyrosine phosphatase (LAR). Using ISH, we found that these CSPG receptors are expressed selectively in poorly regenerating/poorly surviving reticulospinal (RS) neurons both in uninjured animals and after SCI (Zhang et al., 2014a). We also demonstrated by IHC that CSPG immunoreactivities increase significantly at and near an SCI site, peaking at 2 weeks post-TX, and decreasing there after (Zhang et al., 2014a). Enzymatic removal of CSPGs with ChABC soon after SCI rescued many RS neurons from retrograde cell death and enhanced regeneration of their axons. These findings suggested that CSPGs and their receptors may play a role in both the poor intrinsic regenerative ability of some neurons and in their susceptibility to retrograde cell death. However, knockdown of the CSPG receptor PTPσ by morpholinos applied to the SC-TX impaired RS axon regeneration and neuronal survival after SCI (Rodemer et al., 2020). This result was inconsistent with the putative role of PTPσ in axon regeneration and neuronal survival after axotomy. Although several experimental observations suggested that off-target toxic effects of the morpholinos were unlikely, there were other possible explanations for these contradictory results: (1) because there are several receptors binding to CSPGs besides PTPσ (including LAR and Nogo receptors 1 and 3), knocking down one CSPG receptor might induce a compensatory increase in the others. This possibility has not been investigated; (2) CSPGs play key roles in other processes, including guiding axon growth and adhesion during elongation. Guidance of regenerative axon growth may become a priority during recovery from SCI; (3) the knockdown of PTPσ in local cells, e.g., glial cells, at the lesion site may exert indirect effects on the behavior of severed axons.

To understand the distribution and cellular source of lamprey lecticans, we previously cloned a fragment of the most conservative link domain of lecticans from total lamprey CNS cDNA. Using this fragment and ISH, we discovered that lectican mRNAs are broadly expressed in glia and neurons of intact and injured lamprey CNS. To distinguish the expression of the four lamprey lecticans, we obtained probes for lecticans A, B, C, and D (LecA, LecB, LecC, and LecD) from Dr. Daniel Medeiros (Department of Ecology and Evolutionary Biology, University of Colorado, Boulder). In the present report, we investigated the expression patterns of these lamprey lecticans during development and after SCI.

## Materials and Methods

### Animals and Surgery

Lamprey larvae (*Petromyzon marinus*) were purchased from Lamprey Services in Michigan and maintained under an appropriate light cycle in freshwater tanks at 15°C until use. Each year, a few larvae in captivity underwent metamorphosis into adults, and were kept in the same tanks as the larvae. To avoid attrition, they were used as soon as possible for developmental studies. All animal procedures were performed in accordance with a protocol approved by the Temple University Institutional Animal Care and Use Committee (ACUP#: 4492). A total of 43 lampreys were used in the present studies. For SC-TX studies, larval lampreys, 9–11 cm in length (3–4 years old and in a stable phase of neurological development) were anesthetized in saturated benzocaine and pinned to a Sylgard plate filled with ice-cold lamprey Ringer (110 mM NaCl, 2.1 mM KCl, 2.6 mM CaCl_2_, 1.8 mM MgCl_2_, and 10 mM Tris buffer; pH 7.4). The SC was exposed *via* a dorsal incision and completely transected under direct microscopic vision with iridectomy scissors at the level of the 5th gill. After remaining on ice for 2 h to facilitate clot formation, lampreys were returned to freshwater tanks. The animals were allowed to recover at room temperature (RT) for 1, 2, 4, or 10 weeks, then re-anesthetized, and a 1–1.5 cm length of lamprey trunk spanning the TX site was removed, fixed, and processed as below.

### Labeling for CSPGs and PNNs

Body lengths of 1 cm were removed from the gill region and fixed in 4% paraformaldehyde (PFA) for 3–4 h. The specimens were rinsed in phosphate-buffered saline (PBS), dehydrated in serial ethanols overnight in an automatic tissue processor, and embedded in paraffin the next day. To observe whether PNNs surround lamprey neurons, as in mammalian CNS, 10 μm horizontal (parallel to the dorsal surface) sections were cut from the SC, deparaffinized in two changes of toluene (5 min each), rehydrated in a decremental ethanol series (two changes of 100%, 95%, 90%, 80%, and 70%, 5 min each), washed in PBS, and blocked with PBS containing 2% BSA and 0.1% Tween-20 for 1 h at RT. The sections were incubated with biotinylated Wisteria floribunda lectin (WFL, 1:200, Sigma-Aldrich, St. Louis, MO, United States) in blocking solution at 4°C overnight. They were then washed in PBS, blocked as before for 30 min, incubated with alkaline phosphatase (AP)-conjugated streptavid in (1:500, Roche Applied Science, Pleasanton, CA, United States) for 1 h, and washed in PBS followed by SMT buffer (100 mM NaCl, 50 mM MgCl_2_, 100 mM Tris, pH 9.5, 0.1% Tween-20). The chromogenic reaction was carried out in a solution containing 20 μl of NBT/BCIP stock solution (Roche Applied Science) in every 1 ml of SMT on ice in the dark for 10 min or until the reaction was completed. Finally, sections were washed in PBS, dehydrated in serial ethanol solutions, cleared with toluene, and mounted in Permount.

The distribution of CSPGs was studied in the SC of uninjured lampreys. Wholemount or paraffin transverse sections of lamprey SC were immune stained with CSPGs antibodies before and after ChABC treatment. For wholemount IHC, SCs were removed from an anesthetized lamprey in ice-cold Ringer solution *via* a dorsal incision. To improve penetration of the reagents, SCs were treated with a 0.1% solution of type I collagenase (SCR103, Sigma-Aldrich) in lamprey Ringer kept on ice for 10 min, then pinned on a Sylgard-coated plate filled with ice-cold lamprey Ringer. After the *meninx* was stripped away under a dissecting microscope, the SC was transferred, pinned straight on a Sylgard strip, fixed in 4% PFA in PBS for 2–3 h, washed in PBS containing 0.2% Tween-20, blocked in 10% FBS for 1 h, and incubated with an antibody that binds to the glycosaminoglycan (GAG) moieties of all native CSPGs (CS-56, 1:200, Sigma-Aldrich) for 48 h on a nutator at 4°C. The specimens with and without ChABC treatment (see below) were washed thoroughly three times, 1 h each, with PBS containing 0.2% Tween-20, and blocked as above. After the anti-CSPGs antibody was visualized by incubation in fluorescein (FITC)-conjugated anti-mouse IgM secondary antibody (1:200, Sigma-Aldrich) in blocking buffer overnight at 4°C on a nutator, the specimen was washed in PBS and mounted in ProLong Gold antifade reagent (P36934, Invitrogen, Waltham, MA, United States). SC images were captured with a Nikon 80i widefield fluorescent microscope.

To examine CSPGs on paraffin sections, 10 μm transverse sections were deparaffinized in two changes of toluene, 5 min each, and rehydrated in a decremental ethanol series as above. The tissues were washed times in PBS containing 0.2% Tween-20, blocked in the same buffer containing 10% FBS for 20 min, and incubated simultaneously with CS-56 for intact CSPGs and 2B6 (1:200, Seikagaiku, Japan) for digested CSPG stumps overnight at 4°C. Tissues were washed and blocked as above, then visualized by incubation in an FITC-conjugated anti-mouse IgM (1:200, Sigma-Aldrich for CS-56) and Alexa Fluor (AF) 594-conjugated donkey anti-mouse IgG (1:500, Invitrogen for 2B6) secondary antibodies for 1 h at RT. Then slides were washed three times in PBS and mounted in ProLong Gold. ChABC is commonly used to remove side chains of chondroitin sulfate glycosaminoglycans (CS-GAG). To confirm the presence of CSPGs in lamprey SC and the specificity of CSPG antibodies, additional sections were treated with ChABC (C3667, Sigma-Aldrich), 2 mg/ml in enzyme buffer (100 mM Tris, pH = 8.0, 100 mM sodium acetate and 0.02% bovine serum albumin) for 2 h at 37°C, washed and stained for CSPGs with CS-56 and 2B6 antibodies, and images captured as above.

### Identification of Genes for CSPGs in a Lamprey Genomic Database and Polymerase Chain Reaction (PCR) Cloning

The sea lamprey ensemble database and whole-genome sequencing (WGS) trace database maintained by NCBI consists of assembled, partially assembled, and raw unassembled sequencing data from the sea lamprey genome. In preliminary experiments, the sequences of the lectican family from other species, including human, chicken, and zebrafish, were used to query these databases, and four contigs of different lengths were found in the lamprey genomic library using BLAST on NCBI servers, encompassing multiple potential lecticans among the top hits. PCR oligonucleotide primers were designed for the region of the highly conserved link domain based on these contig sequences. Total RNA from lamprey CNS was isolated using Trizol reagent (Invitrogen). The first-strand cDNA synthesis reaction from total RNA was catalyzed by Superscript III Reverse Transcriptase with oligo-dT or 8 bp random primers. The synthesized total cDNA served as templates for PCR cloning using the Expand^TM^ High Fidelity PCR System (RocheApplied Science) as per the manufacturer’s protocol. A PCR product of approximately 420 bp has been yielded. This was purified on 1% agarose gel, ligated into the pGEM-T Easy Vector (Promega, Madison, WI, United States), sequenced (GENEWIZ, South Plainfield, NJ, United States), analyzed by BLAST, and as expected, found to be in the highly conserved lectican link domain. For this reason, the specific lectican identity could not be defined, but this fragment served as a template to synthesize a probe (probe 1) for investigation of lamprey lecticans nonspecifically.

### Lamprey Lectican Family and Phylogenetic Analysis

Eleven gene sequences of the lamprey lectican family have been published recently and annotated at NCBI (annotation release 100). The sequences were derived from lamprey genomic sequences, predicted by automated computational analysis, and annotated using the Gnomon gene prediction method developed at NCBI. Among these 11 lamprey lectican sequences, six were annotated as ACAN variants; three were VCAN variants, one of which had a very different sequence from the other two, and two were NCAN variants (see Supplementary Figure 1 for alignment analysis of the lectican family). No BCAN were annotated. However, the sequences of four lamprey lecticans were assembled recently from transcriptomic reads of t.26.5 embryos (Tahara, 1988) and late larval oral disc tissue (Root et al., 2021). Based on studies of phylogeny and genomic arrangement, it was concluded that lamprey lecticans lack clear one-to-one orthologies to gnathostome lecticans, and therefore, the lamprey lecticans were designated simply as“A”, “B”, “C”, and “D”. Our own alignment analysis indicates that these four lecticans are only partial sequences of the lamprey lecticans annotated by NCBI Gnomon (Supplementary Figure 2). Therefore, in the current study, the gene peptide sequences annotated by NCBI corresponding to lecticans A, B, C, and D were used for phylogenetic analysis, using the unweighted pair group method with arithmetic mean (UPGMA). The NCBI lectican peptide sequences of other species and their accession numbers are listed in Supplementary Table 1.

### Riboprobes

The non-specific lectican probe 1 (see above) covers nucleotides 385–800 (416 bp) in the open reading frame of the previously annotated lamprey ACAN (L-ACANx1; XM_032973693). The cDNA probe templates for lecticans A, B, C, and D, generously provided by Dr. Medeiros’ lab (Root et al., 2021), had target gene open reading frames annotated by NCBI Gnomon as follows: probe for lectican A (LecA), 674–1,207 (534 bp) of L-VCAN x1 (XM 032949615); probe for lectican B (LecB), 1,132–1,681 (550) of L-ACAN x3 (XM 032973695); probe for lectican C (LecC), 10,562–11,111 (550 bp) of L-VCAN (XM 032968631); and probe for lectican D (LecD), 3,634–4,183 (550 bp) of L-NCAN x1 (XM032975972). The locations of each probe underlined on the domain structure of their corresponding gene, and the percent identities of each probe with other lecticans are given in Supplementary Figure 3. Percent identity was restricted to the aligned region, which was determined by the alignment of each probe sequence with the other three lectican sequences, respectively, using Vector NTI (Life Technologies, Carlsbad, CA, United States). For rapid generation of a template for riboprobe synthesis, the T7 promoter sequence was added to the 5’ end of each antisense primer. The digoxigenin (Dig)-labeled antisense RNA probes were constructed from amplified cDNA templates, which contained the T7 promoter sequence upstream of the antisense-strand, using an RNA transcription kit with Dig-labeling Mix (Roche Applied Science) as per the manufacturer’s instructions.

### Developmental Study

To investigate developmental changes in the mRNA expression of each lectican, ISH was carried out on an embryo at stage t.28 (a gift from Dr. Anton Barreiro-Iglesias at the University of Santiago de Compostela, Spain), larval lampreys from 3.5 to 13 cm in length (one each of 3.5, 5, 8, 9.5, and 13 cm), and a recently transformed adult (14 cm). After lampreys were anesthetized by immersion in saturated aqueous benzocaine until motionless to tail pinch, 1 cm tissue blocks were removed from the gill region, fixed in 4% PFA in PBS for 3–4 h at RT, rinsed in PBS, dehydrated in serial ethanols overnight in a tissue processor, and embedded in paraffin. A pre-fixed whole embryo sample was embedded as well. To reduce the variations caused by different animals and the number of animals used, 10 μm serial sections were cut from each block and alternately mounted on four glass slides for ISH with each lectican probe: LecA, LecB, LecC, and LecD (see below).

### *In situ* Hybridization

ISH was performed on 10 μm paraffin sections of non-lesioned lamprey brain, rostral SC, and SC spanning the TX site at 1, 2, 4, and 10 weeks post-TX. The sections were deparaffinized and rehydrated in a decremental series of ethanol as above. They were then washed in PTW (0.1% Tween-20 in PBS, 3 × 5 min), and pre-hybridized at 50–55°C for 1 h in hybridization solution [50% deionized formamide, 5× standard saline citrate (SSC), 100 mg/ml Torula yeast RNA, 100 mg/ml wheat germ tRNA, 50 mg/ml heparin, 0.1% Tween-20]. The Dig-probes for lectican (probe 1, LecA, LecB, LecC, and LecD) were applied to slides at a concentration of 2 μg/ml in a hybridization solution and incubated overnight at 55°C. To prevent evaporation, the slides were covered with HybriSlip (Sigma-Aldrich) and kept in a moist chamber. On the next day, the slides were washed in hybridization solution at 55°C (3 × 10 min), PTW/hybridization solution (1:1) for 10 min, PTW at RT (3 × 10 min), and PBT (0.1% bovine serum albumin, 0.2% Triton X-100 in PBS, 3 × 10 min). AP-conjugated Dig antibody diluted 1:1,000 in PBT was applied overnight at 4°C. The sections were then washed in PBT and SMT (each for 3 × 5 min), and the chromogenic reaction was performed in AP substrate NBT/BCIP as above for PNNs staining. The reaction was stopped in PBS, and the slides dehydrated, cleared, and mounted in Permount. The distribution of lectican mRNA in lamprey CNS was determined earlier using probe 1 and four different lectican probes, LecA, LecB, LecC, and LecD were used to distinguish their mRNA expression in SC during development and after SCI.

### Double Labeling of Lectican mRNA With Cell Marker Antibodies

To determine the identity of lectican mRNA-positive cells, combined fluorescent-labeling of ISH and IHC was performed on cross-sections of non-injured SC and horizontal sections spanning the TX-site. After overnight hybridization with Dig-probes (probe 1), the sections were washed and incubated with either the monoclonal anti-Hu antibody for all neurons (1:100, Molecular Probes) or LCM29 (1:100, lamprey glia antibody), overnight at 4°C. On the following day, after three washes in PBS the sections were visualized by AF488-conjugated anti-Dig for ISH labeling and by AF594 donkey anti-mouse secondary antibody IgG for anti-Hu or LCM29 antibodies. The sections were again washed in PBS 3 × 5 min, then mounted in ProLong Gold antifade reagent, observed, and imaged using a Nikon 80i microscope.

Because of the higher auto-fluorescent background in reactive tissues after injury, to investigate the type of lecticans-producing cells in the TX-site, the ISH and IHC labeling were also demonstrated colorimetrically using chromogenic substrates. Ten μm horizontal sections spanning the lesion site were cut at 1, 2, 4, and 10 weeks post-TX and double labeled by ISH for lectican mRNAs and IHC for glia and neurons, as above. Following overnight Dig-probe hybridization, the sections were incubated with AP-conjugated Dig (1:1,000, Roche Applied Science) and LCM29 or anti-Hu antibodies overnight at 4°C. The next day, the LCM29 or anti-Hu antibodies were detected by VECTASTAIN Elite ABC Universal Plus, Peroxidase (HRP) Kit (PK-8200, Vector Laboratories, Newark, CA, United States) following the protocol provided by the manufacturer. Finally, the sections were visualized by a blue color, using BCIP/NBT (Roche Applied Science) substrate for AP, as above, and, after washing ×3 in PBS, by a brown color, using metal-enhanced diaminobenzidine (DAB, Thomas Scientific, Swedesboro, NJ, United States), a chromogenic substrate for HRP. The reactions were stopped in PBS, and the slides dehydrated, cleared in two changes of toluene, and mounted in Permount. The isolectin B4 (IB4) of *Griffonia simplicifolia* is widely used in different species, including lamprey (Shifman and Selzer, 2007; Rodemer et al., 2020), as a microglia marker, because it binds to D-galactose residues in both resting and activated microglial cells (Streit, 1990). In addition to the LCM29 and anti-Hu antibodies, the lectican-producing cells were also doubly stained with IB4–HRP (5 μg/ml in PBS, Sigma–Aldrich, St. Louis, MO, USA, Cat# L5391) overnight using DAB histochemistry, as described above.

### Quantification and Statistical Analysis

The results of ISH on horizontal sections showed that the four lamprey lecticans have similar expression patterns in the uninjured SC, but that expression levels differ before and after SCI. It is difficult to quantify staining on horizontal sections because the cutting plane and SC borders differ from section to section. To quantify the expression level of each lamprey lectican, transverse sections of SC were prepared from uninjured lampreys and at 1, 2, and 4 post-TX. Ten μm serial sections were cut rostralward from the TX-site or from the level of the 5th gill in uninjured animals, mounted alternately on four slides, and processed for ISH with probes of LecA, LecB, LecC, and LecD respectively, as above. SC images (5–10 sections per animal) were captured using a Nikon 80i widefield microscope. All these transverse sections were outlined and staining in surrounding tissues (muscles, notochord, and meninges) was removed by Adobe Photoshop (CS4). Semi-quantitation was performed using image analysis software ImageJ (1.52a). First, the image was converted to an 8-bit black and white format, and the SC area of each animal was determined (in pixels) after adjusting the threshold so that the entire SC became completely dark. The ISH-stained area of each section was then measured after adjusting the threshold to the point that the image pattern became identical to that of the actual histological staining (in binary pixels). The ratio between ISH labeling and the SC area was defined as “percent intensity” for each image. Statistical analysis was performed on the mean intensity for each lectican. After analysis of the normality and homogeneity of the variance, a parametric or non-parametric statistical instrument was selected. Statistically significant differences among the groups were analyzed by one-way analysis of variance (ANOVA), followed by the Tukey or Dunnett’s multiple comparisons test respectively. Animal numbers are indicated in the individual figure legends. All values were expressed as mean ± SEM.

## Results

### CSPGs in Lamprey SC

**Figure 1:**
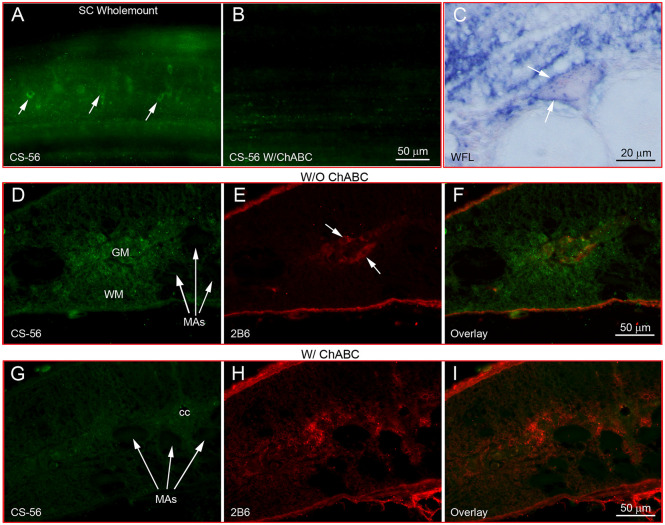
CSPGs are present in lamprey spinal cord and contribute to perineuronal nets. **(A)** Labeling of CSPGs with antibody CS-56 in SC wholemount shows a perineuronal distribution (arrows). **(B)** Loss of CSPG labeling after treatment with ChABC. **(C)** Labeling of lectins by WFL in a transverse section of SC shows the ring structure of PNNs (arrows). **(D–F)** CSPG immunofluorescence in transverse sections of SC without treatment with ChABC. **(D)** Intact CSPGs labeled by CS-56. **(E)** Sparse staining of CSPG stumps with antibody 2B6. **(F)** Overlay of **(D)** and **(E)**. **(G–I)** As in **(D–F)** but after treatment with ChABC. CSPGs were broadly distributed in gray matter (GM) and white matter (WM) of normal lamprey SC, but not inside the large Müller axons (MAs). A few SC neurons were labeled by 2B6 before ChABC treatment (arrows in **E**), indicating the neuronal distribution of unsaturated disaccharide CSPG core proteins. ChABC treatment dramatically reduced the CS-56 immunoreactivity **(G)**, while staining for CSPG stumps was increased **(H)**. **(I)** Overlay of **(G)** and **(H)**.

Wholemount IHC showed that CSPGs were widely distributed along the entire SC, especially in and around neurons (Figure 1A). Digestion with ChABC eliminated the labeling (Figure 1B). WFL staining in horizontal sections also revealed lectin reactivity around neurons (Figure 1C). These images resemble the staining pattern of PNNs described in mammalian CNS (white arrows, Figures 1A,C). On transverse sections of SC without ChABC treatment, reactivity to CS-56 antibody, which recognizes GAG moieties of native CSPGs, appeared in the extracellular matrix (WM), as well as in cell bodies of the gray matter (GM, Figure 1D). Some neurons were labeled by 2B6, an antibody recognizing digested CSPG stumps (arrows, Figure 1E). Digestion with ChABC greatly reduced CS-56 staining (Figure 1G) while increasing the staining for 2B6 (Figure 1H), confirming the specificities of CS-56 and 2B6 antibodies and the presence of CSPGs in lamprey SC, as in other species.

### Neurons and Glia Express Lecticans in Uninjured Lamprey CNS

**Figure 2:**
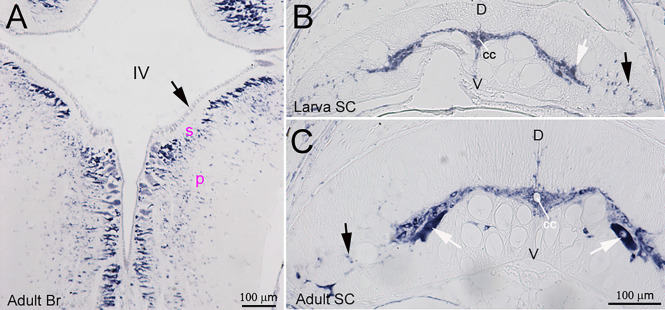
Distribution of lectican mRNAs in lamprey CNS. ISH using probe 1 shows broad expression of lectican mRNAs in lamprey brain and spinal cord. **(A)** A horizontal section of an adult brain (Br) shows that lectican mRNAs are expressed in neurons and glia but only weakly in the ependyma (arrow), IV = 4th ventricle; s = subventricular zone; p = peripheral zone. Transverse sections of spinal cord (SC) from a larva **(B)** and an adult **(C)** show that lectican mRNAs are expressed in SC neurons (white arrows) and glia (black arrows) and that the level of lectican mRNAs is higher in adult SC than in larval SC. D = dorsal; V = ventral; cc = central canal.

**Figure 3:**
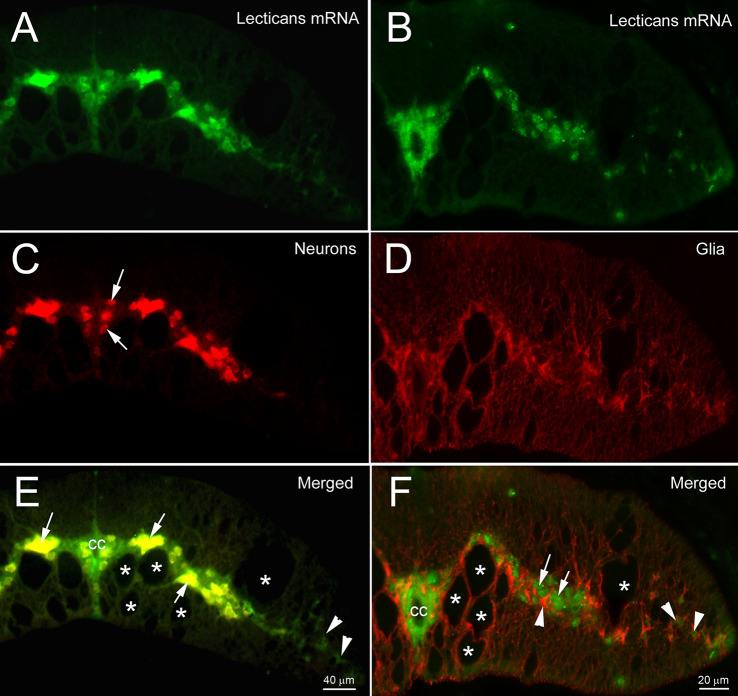
Expression of lectican mRNAs in neurons and glia. Transverse sections of lamprey spinal cord are double labeled by lectican ISH, using probe 1 **(A,B)** and by anti-Hu, an antibody specific for neuronal cell bodies **(C)**, or LCM29, an antibody for glial keratins **(D)** that labels glial cell bodies and processes. Arrows in **(C)** point to cerebrospinal fluid (CSF)-contacting neurons. **(E,F)** Overlayed images show the expression of lectican mRNAs in neurons (arrows in **E** and **F**) and glia (arrowheads in **E** and **F**). cc = central canal. Asterisks = giant axons (Müller axons in the ventromedial white matter and the more dorsolateral Mauthner axon).

Lectican mRNAs are expressed broadly in lamprey CNS. ISH with the previously synthesized probe 1 showed that, in a young adult brain, all the neurons and glial cells located in the subventricular zone (s, Figure 2A) and peripheral region (p, Figure 2A) expressed lectican mRNAs, but that the ependyma surrounding the fourth ventricle was labeled only lightly or not at all (arrow, Figure 2A). In the SC, the positive cells included large neurons in gray matter (white arrows, Figures 2B,C) and small cells around the central canal (cc) and in the lateral white matter (black arrows, Figures 2B,C). Lectican-positive neurons and glia were identified by combined ISH and IHC on transverse sections of uninjured lamprey SC (Figure 3). Lectican-positive cells labeled in green by fluorescent ISH were mainly distributed in a ribbon-shaped expanse of gray matter extending laterally from the central canal (Figures 3A,B). Hu-antibody labeled all the spinal neurons, including some small cerebrospinal fluid (CSF)-contacting neurons surrounding the central canal (arrows, Figure 3C). Some laterally located small cells were labeled by the lectican probe but not by the Huantibody (arrowheads, Figure 3E). Their locations and staining patterns suggest that they are most likely glia. All ventrally located giant Müller axons and the dorso-laterally located Mauthner axons were unlabeled (asterisks, Figure 3E). Small cells in gray matter and cells near the lateral edge of SC were labeled by the glial antibody marker LCM29 (Figure 3D) and by ISH (arrowheads, Figure 3F). LCM29 also labeled glial processes, but not giant Müller axons (asterisks, Figure 3F) or neurons, which were labeled only by the lectican probe (arrows, Figure 3F). These results confirmed the specificity of the antibodies and showed that lamprey lecticans are expressed in both neurons and glia.

### Lamprey Genome Has Homologs of Vertebrate Lecticans

**Figure 4:**
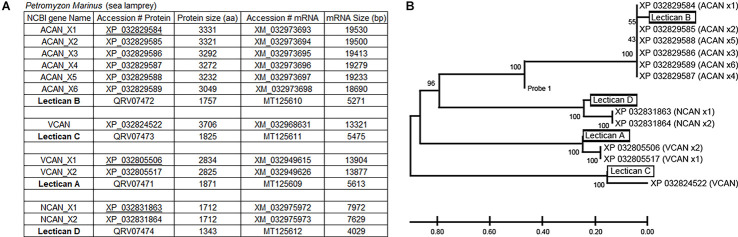
Analysis of lamprey lectican family. **(A)** A table summary of the lamprey lectican family found in the NCBI database. The protein sequences are predicted by automated computational analysis of the sequenced lamprey genome, annotated using the Gnomon gene prediction method and named as ACAN: Aggrecan; VCAN: Versican; NCAN: Neurocan. X represents a variant of each family member. The alignments of the variant sequences of each member are shown in Supplementary Figure 1. The sequences of lecticans A, B, C, and D (in bold) are published in NCBI and by Root et al. (2021). They are assembled from four different lamprey genomic scaffolds containing exons with sequence similarities to those of jawed vertebrate lecticans. The designation of lamprey lecticans as A, B, C and D is due to the lack of a clear one-to-one orthology with gnathostome lecticans. **(B)** Phylogenetic analysis shows the identities of lamprey lecticans, A, B, C, and D, which are highlighted in boxes and grouped in the table **(A)** with their closest homolog. However, alignments show that lectican A, B, C, and D are only partial and discontinuous sequences that are found in their homologous lecticans annotated by Gnomon (see Supplementary Figure 2).

**Figure 5:**
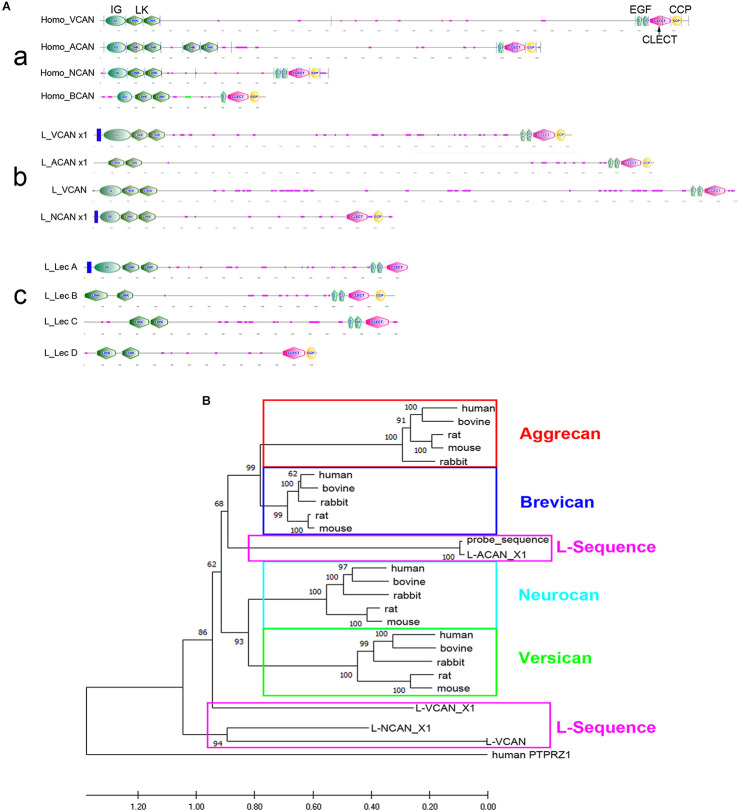
Structural comparison and molecular phylogeny of vertebrate lecticans. **(A)** Domain structures of human (**a**) and lamprey lecticans (**b,c**) with the N-terminus to the left. IG = Immunoglobulin-like domain; LK = link domains; EGF = EGF-like domains; CLECT = C-type lectin domain, also known as the carbohydrate-recognition domain (CRD); CCP = complement control protein domain. The orders of each lamprey lectican annotated by Gnomon (**b**) correspond to lectican A, B, C, and D (**c**). **(B)** Phylogenetic relationships of mammalian and lamprey lecticans based on amino acid sequence alignments. Lamprey sequences are in pink boxes; the individual members of the mammalian lecticans are in other colored boxes. Maximum likelihood analysis scores are shown at the respective nodes. Human PTPRZ1 sequence is designated as an outgroup. The accession numbers for all sequences can be found in Supplementary Table 1.

Axons of the identified RS neurons of lampreys vary in the probability that their axons will regenerate across the TX-site (Jacobs et al., 1997). Those that are bad regenerators also tend to undergo delayed retrograde neuronal death after axotomy (Shifman et al., 2008). Enzymatic digestion of CSPGs with ChABC increased the probability of regeneration and reduced apoptotic signaling in RS neurons (Hu et al., 2021). We also cloned the CSPG receptors, PTPs and Lar, and found that they are selectively expressed in bad regenerators (Zhang et al., 2014a). These findings support a role of CSPGs in axonal regeneration and/or retrograde neuronal death and raise the question of whether specific lecticans play different roles in neuronal survival and axon regeneration. All four lecticans—NCAN, BCAN, VCAN, and ACAN—share similar N-terminal and C-terminal domains, with various chondroitin sulfate chains in the central domain. In the present study, after searching the non-assembled and partially assembled lamprey genomic databases (see “Methods” Section), we used the sequences of highly homologous domains from various species to identify four contigs of lamprey lecticans (GL478684, GL486137, GL477982, and GL486820) with lengths of 5,502, 6,289, 459, and 567 bp, respectively but none of these were full-length sequences, and BLAST analysis failed to clearly delimit them to specific lectican species, although the 5’ prime link domain was included in the first three contigs. PCR cloning using primers designed from the link domains of the three contigs resulted in a fragment from the total lamprey CNS cDNA of th expected size (~420 bp) of contig GL478684. This cloned cDNA fragment served as a probe template in our early experiments. More recently, our search of the NCBI database revealed several newly released predicted sequences of the lamprey lectican family by the NIH’s computational analysis, which were annotated in relation to the mammalian lectican family. Their names and access information are listed in Figure 4A. According to BLAST, the contigs of GL478684, GL486137, GL477982, and GL486820 found previously represent the annotated lamprey versions of ACAN, a VCAN variant, VCAN, and NCAN, respectively. Supplementary Figure 1 shows the aligned amino acid sequences of the variants of each lectican. The six variants of lamprey ACAN (L-ACAN; Supplementary Figure 1A) and two variants of L-VCAN (Supplementary Figure 1B) may be derived from alternative splicing or produced by computational error. The one annotated as L-VCAN is very different from the other two variants ofVCAN (Supplementary Figure 1C), and may belong to a fourth member of the lectican family. Two L-NCAN variants differ at the mRNA sequence level but are 100% identical at the protein level (Supplementary Figure 1D). Because phylogenetic analysis failed to conclusively support a one-to-one orthology between lamprey and gnathostome lectican paralogs, the lamprey lecticans have recently been named simply A, B, C, and D (Root et al., 2021). We carefully analyzed the relationship among all published lamprey lecticans and list them in Figure 4. The sequences published by Root et al. (2021) are partial and discontinuous compared to the sequences predicted by NIH. The alignments of lecticans A, B, C, and D with each of their corresponding NIH-designated lectican sequences are shown in Supplementary Figure 2. We analyzed the protein domains of all lamprey lecticans found in NCBI using the Simple Modular Architecture Research Tool (SMART, V 9.0) and compared them with human lecticans (Figure 5A). The sequences of lecticans A, B, C, and D are much shorter than the sequences predicted by NIH. However, the major archetypal domain structures of lectican proteins are present because the missed sequences are in the middle of the gene, which does not contain any conserved domains. As reported (Root et al., 2021), no lamprey lectican possessed the extra link domain seen in human and other mammalian ACANs, even using the full-length lectican genes predicted by NIH. However, the complement control protein (CCP) domain, which is reported missing in lectican A by Root et al. (2021), appeared in its corresponding longer sequences of lamprey VCAN variants.

When the probe sequence of LecB is aligned with that of lectican B published by Root et al. (2021), and with those of all variants of L-ACAN published on the NCBI website, only L-ACAN_x3 is 100% aligned; all the others have a 117 bp gap in the middle (LecB missed 117 bp). To confirm the accuracy of the LecB sequence and that of the LecB targeted L-ACAN variant present in lamprey CNS, we performed PCR cloning using primers designed from the 5’ and 3’ prime portions of the LecB sequence. A λZap cDNA library of lamprey CNS was used as a template. PCR yielded a single band at ~500 bp. The sequence of the PCR product was 100% identical to the LecB sequence (data not shown), confirming that the LecB-targeted L-ACAN x3 is the one existing in lamprey CNS.

Phylogenetic analyses using lectican protein sequences from selected species (Supplementary Table 1) divided the four lectican members into ACAN + BCAN and VCAN + NCAN subfamilies (Figure 5B, as per the findings of Root et al. (2021). Our phylogenetic analyses with the full-length lectican genes published by NIH in NCBI also showed that none of the lamprey lecticans consistently grouped within any of the four gnathostome lectican paralog groups. These results were consistent with the findings of Root et al., 2021, but our analyses placed the L-ACAN variants (corresponding to lectican B) in the ACAN + BCAN subfamily, which differs from their placement of lectican A in the ACAN + BCAN subfamily. The other three lecticans were like the VCAN + NCAN subfamily (Figure 5B).

### Developmental Expression of Lectican mRNAs in Lamprey CNS

**Figure 6:**
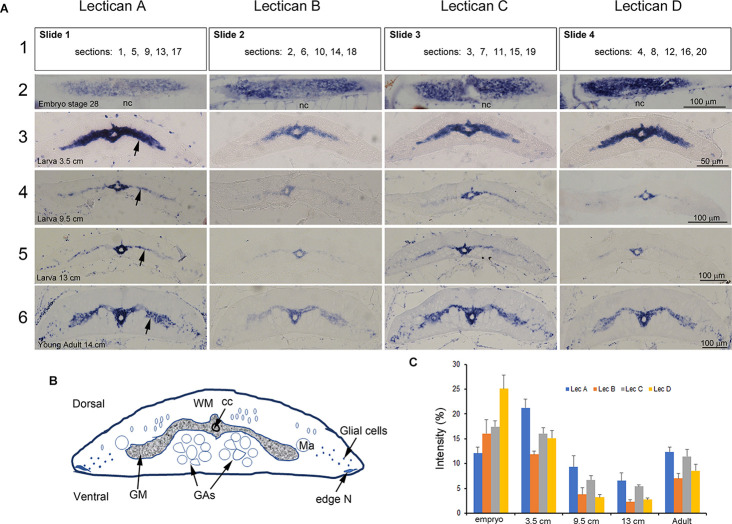
Developmental expression of lamprey lecticans in the spinal cord. **(A)** ISH of lecticans in lamprey at different ages. Serial longitudinal (embryo only) or transverse sections from each animal’s SC at the level of the 5th gill were mounted alternately on four slides, as shown in the top row (row 1). Numbers in the rectangles present the order of the sections. ISH was performed on each of these slides using four different probes, LecA, LecB, LecC, and LecD, respectively. The developmental stage or size of the lamprey is indicated on the sections in column 1. The sections in each image are representative for that animal at that level, and thus are comparable between lecticans. Note that in the larva, lectican D predominated, whereas in the larval and adult stages, lecticans A and C predominated. The expression levels of all lecticans were relatively high at early larval stages, declined at later larval ages, and then increased again after metamorphosis to the adult stage. nc = notochord. (**B)** A cartoon showing the structure of a transversely sectioned lamprey SC. Ma = Mauthner axon; GAs = Giant axons; WM = white matter; GM = gray matter; N = neuron; cc = central canal. **(C)** Semi-quantification of staining intensity shows the changes in the four lecticans during development. “Intensity (%)” = 100 × the ratio between the number of pixels that are labeled by ISH, and the total number of pixels encompassed within the SC perimeter (see “Methods” Section).

Both neuron-intrinsic and -extrinsic factors have been invoked to explain the developmental reduction of regenerative capacity in the CNS (Yun, 2015). Even in the adult lamprey, which shows substantial regeneration of RS axons (Lurie and Selzer, 1991a), regeneration is less robust than in larvae (Cohen et al., 1989). To the possibility that this reflects developmental changes in lectican expression, we measured lectican mRNA levels in lamprey CNS at different developmental stages, from embryo to newly transformed young adult. Expression of lectican mRNAs was intense in embryonic and early larval (<5 cm, 1–2 years old) lamprey SC, declining at later larval stages, and increasing again just before metamorphosis and into the young adult stage (Figures 6A,B). All lecticans showed a similar staining pattern, which is quantified in Figure 6C. The expression patterns of all four lecticans were similar in spinal cord neurons and glia, although the levels of expression differed. In uninjured larval lampreys 8–13 cm in length (4–5 years old), lecticans A and C were dominant (Figure 6A). Following SCI, however, lecticans B and D predominated, although all lecticans were upregulated early after injury (see below).

### Increased Expression of Lecticans Following SCI

**Figure 7:**
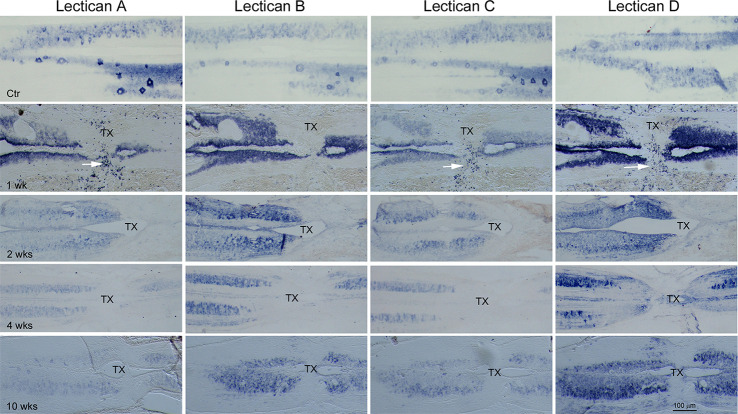
The expression of lecticans after SC-TX. ISH using probes LecA, LecB, LecC, and LecD, individually, performed on horizontal sections of non-injured (Ctr) or injured animals spanning a TX-site mounted alternately on four slides as described in Figure 6. After SC-TX, robust upregulation of lecticans B and D causes them to predominate over lecticans A and C, which dominated before TX. Upregulation of lectican D, in particular, persisted at least 10 weeks post-TX. Many small cells in the TX site are densely labeled by LecA, LecC, and LecD, but not by LecB, at 1 week post-TX (white arrows). IB4-HRP staining identifies only a few of these small cells as microglia/macrophages (see Figure 9). These results suggest that lecticans B and D may play important roles after SC injury. TX = transection site.

**Figure 8:**
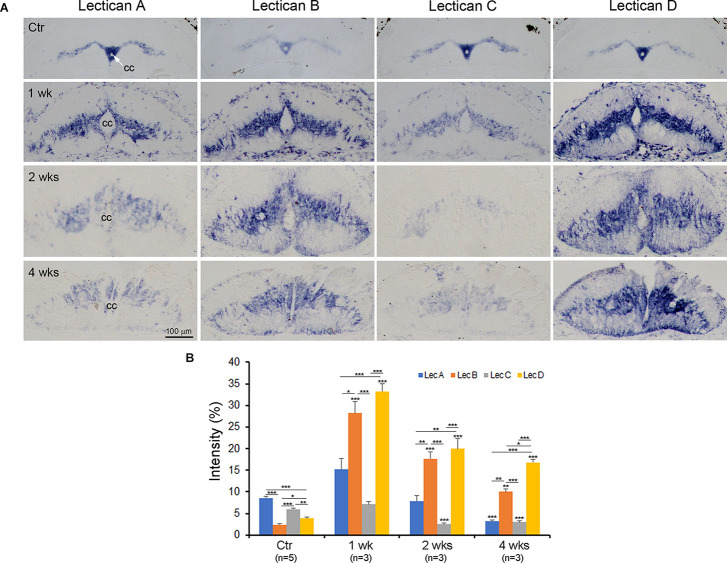
Time course of lectican expression after SC-TX. To quantify the changes of the four lecticans after SCI, transverse sections just rostral to the TX were mounted alternately on four slides as described and ISH performed using probes LecA, LecB, LecC, and LecD, respectively **(A)**. As in the horizontal sections, compared to non-injured SC (top row), all four lecticans are upregulated by 1-week post-TX, then gradually decline. Lectican A and C return to levels near or lower than those of ctr SC at 2 weeks, but levels of lectican B and D remain higher than control through all time points investigated. The increase in lectican D is the most marked. The central canal (cc) is expanded after SC transection. **(B)** Semi-quantification of staining intensity shows the changes in the four lecticans after SC transection. n = the number of animals in each group. Five to eight sections from each animal were analyzed. Error bars indicate SEM. The intensity level of each lectican after SCI is compared with its level in control SC and within the group in each time point. Significance probabilities: *p < 0.05; **p < 0.01; ***p < 0.001.

**Figure 9:**
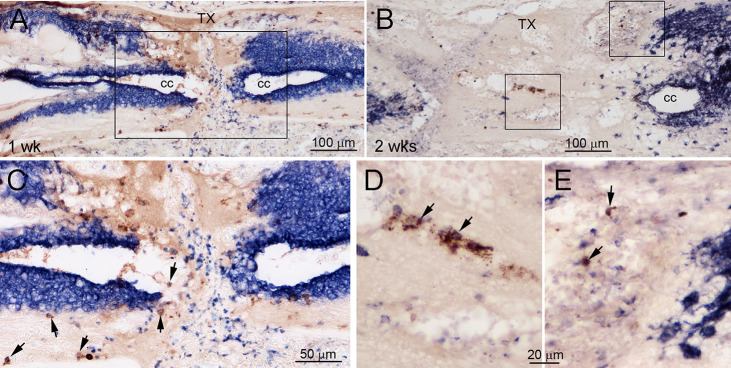
Co-expression of lectican mRNA and a marker of microglia/macrophages. To characterize the small lectican-positive cells appearing early after TX, horizontal sections spanning the TX site were labeled colorimetrically with IB4-HRP after lectican ISH at 1 week **(A,C)** or 2 weeks **(B,D,E)** post-TX. **(A)** The section at 1 week from Figure 7; **Lectican D**, now labeled by IB4, demonstrates that most of the small cells at the TX site that are lectican-positive by ISH do not label for IB4, most likely because they are blood cells or fibroblasts derived from surrounding tissues. **(C)** Enlargement of the rectangle indicated in **(A)**. Arrows point to doubly labeled cells. **(B)** A section at 2 weeks post-TX shows a reduced number of small cells positive for lecticans by ISH in the TX site and that some bind IB4. **(D,E)** Enlarged images of squares indicated in **(B)**. Arrows point to doubly labeled cells. cc = central canal.

The lamprey CNS contains both CSPGs and their receptors. CSPG levels increase near the TX, peaking at 2 weeks post-TX and returning to normal by 10 weeks (Zhang et al., 2014a). Digestion of CSPGs with ChABC promotes axon regeneration and prevents retrograde RS neuron death after SCI (Hu et al., 2021). Whether specific lecticans are involved is not known. Therefore, we performed ISH with probes for the four lamprey lecticans at different times between 1 and 10 weeks after SCI. We examined horizontal SC sections spanning the lesion site qualitatively (Figure 7), and used transverse sections near the lesion site for quantitative analysis (Figure 8). SCI increased the expression of all four lectican mRNAs. Interestingly, whereas lectican A and C were dominant in uninjured SC, lectican B, and D became dominant in and around the TX-site. The increases in expression peaked in the first week post-TX, then declined. Expression of lecticans A and C declined more dramatically than B and D, and lectican D remained upregulated at all time points observed. The expression of each lectican during recovery from SCI mimicked that found in the embryo (Figure 6, row 2). In the first week, many small cells at the injury site were labeled by probes for lectican A, C, and D, but not lectican B; these are most likely blood cells. IB4 labeling identified some of these cells as microglia/macrophages (Figure 9).

Chromogenic IHC staining intensity is non-linear, and thus not suitable for quantitative analysis. Unfortunately, quantitation of fluorescence imaging near an SCI is hindered by high background fluorescence. Therefore, we performed semiquantitative lectican expression using the Limit to Threshold method with Image J (Fuhrich et al., 2013; Crowe and Yue, 2019; Jin et al., 2020). Horizontal sections are not convenient for this purpose because the lamprey spinal cord is relatively flat, and alternately mounted sections from the same animal are not comparable in overall area, nor in the amount of gray matter they contain. Therefore, we performed ISH on transverse sections, as shown in Figure 8, although staining patterns were consistent with the results obtained in horizontal sections. Expression of lectican mRNAs peaked in the first week post-TX, then declined. Lectican D expression was increased the most of the four lecticans, and remained elevated at all time points observed (Figure 8A). Semi-quantification (Figure 8B) showed that in uninjured SC, staining intensities of lecticans A and C were significantly higher than those of lecticans B and D, but with the dramatic increases in lectican B and D staining after SCI, this relationship was reversed. Expression levels of lectican A and C were only slightly elevated at 1week after SCI, and then significantly decreased.

### Cellular Sources of CSPGs in the “Glial Scar”

CSPGs upregulated after SCI in mammals are generally assumed to be secreted by reactive astrocytes (Silver and Miller, 2004; Busch and Silver, 2007; Galtrey and Fawcett, 2007). In lamprey, SC-TX causes glial processes that are normally arranged transversely to reorient longitudinally, forming a scaffold for axonal growth across the TX. Whether this glial reaction should be called a “scar” is debated, since it is not hard, like scars in mammals, and specifically supports axon growth (Lurie and Selzer, 1991b). As in mammals, CSPG levels are upregulated after SCI in lampreys, peaking at 2 weeks, and then declining. To determine the cellular sources of the CSPGs, we combined ISH with probe 1 (belonging to lectican B) or LecD with histochemistry using a marker for microglia/macrophages (IB4-HRP), or IHC using markers for neurons (anti-Hu), and glia (anti-glial keratin antibody LCM29). Early after SCI, many small lectican-positive cells appeared in the TX site (top row, Figure 7). Only some of these small lectican-positive cells were labeled by IB4 (arrows in Figure 9C). At 2 weeks post-TX, there were fewer lectican-positive cells in the core of the injury site, only some of which were doubly labeled by IB4 (Figures 9B,D,E). These results indicate that many other lectican-positive cells besides immune cells invade the injury site early after SCI.

**Figure 10:**
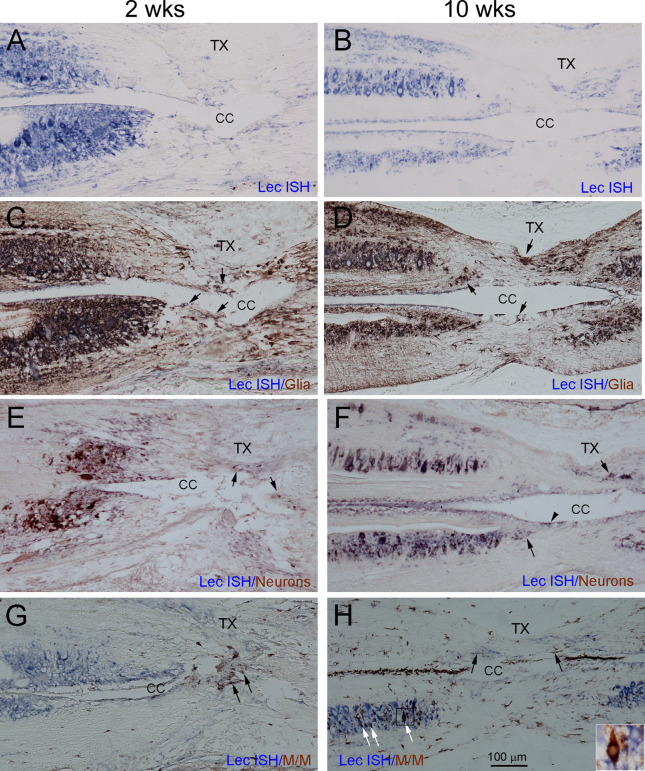
Glia, neurons, and microglia/macrophages all contribute to the increase in CSPGs after SCI, and to scar formation. To characterize the cellular sources responsible for the increase in CSPGs after lamprey SCI, serial horizontal sections spanning the lesion site were collected from each lamprey at 2 (left column) and 10 weeks (right column) post-TX and processed by ISH (in blue) to detect lectican mRNAs, using probe 1. After ISH, IHC was performed (in brown) to identify glia **(C,D)**, neurons **(E,F)**, and microglia/macrophages **(G,H)**. **(A,B)** Sections labeled by ISH alone show expression of lectican mRNAs primarily in gray matter around the central canal, and to a more limited extent, in the lesion site. **(C,D)** The same sections shown in **(A,B)** are additionally stained for glia. Arrows point to doubly labeled glial cells in the lesion site. **(E,F)** The same sections show doubly labeled neurons in the lesion site (arrows). Arrowhead in **(F)** indicates CSF-contacting neurons (Zhang et al., 2014b). **(G,H)** The same sections show IB4-positive microglia/macrophage profiles in the lesion site; some are doubly labeled by lectican-ISH (black arrows). At 10 weeks **(H)**, the numbers of IB4-positive microglia/macrophage profiles are increased, and some neurons also are labeled by IB4 (white arrows). The inset in **(H)** is an enlarged image of the small box showing a neuron doubly labeled by ISH and IHC.

Although fluorescence ISH and IHC are the most straightforward ways to colocalize the expression of mRNA and proteins, the experiments are complicated by increased auto-fluorescence at the injury site. Commercially available auto-fluorescence suppression kits can also quench true signals. To eliminate confusion due to auto-fluorescence, we performed double chromogenic lectican ISH with IHC, using markers for glia, neurons, and microglia/macrophages. Figure 10 shows horizontal sections spanning the TX site taken from animals at 2 weeks and 10 weeks post-TX, and stained for different markers. Cells doubly labeled by lectican-ISH and IHC using the neuronal or glial markers were located primarily outside the core of the glial reaction. Relatively few lectican-positive cells inside the “scar” were doubly labeled by glial (arrows, Figures 10C,D) or neuronal (arrows, Figures 10E,F) markers. The core of the scar at 2 weeks had more cellular elements than at 10 weeks. The small sizes of the cells labeled by the neuronal marker in the core region (arrows in Figure 10E) suggested that they were most likely CSF-contacting neurons that are located in the subependymal region surrounding the central canal in the intact SC (Chiba et al., 1981; Rodicio et al., 2008; Villar-Cervino et al., 2008; Zhang et al., 2014b; Jalalvand et al., 2018). The non-neuronal (ependymal glia) cells were much less heavily labeled. We also performed fluorescence double labeling to confirm the chromogenic staining. Supplementary Figures 4, 5 illustrate glia and neurons, respectively. At 2 weeks, the glial processes (white arrowheads, Supplementary Figures 4C,G,I) were arranged in parallel with axons (black arrowheads in Supplementary Figures 4I,J) and ran toward the lesion site but had not yet invaded the center of the lesion. By 4 weeks, glial processes completely spanned the lesion site (white arrowheads, Supplementary Figure 4J) and comprised the major component of the “scar”, creating a potential bridge for regenerating axons traversing the injury site. Although a few lectican-positive glia were present in the center of the “scar” (white arrows, Supplementary Figure 4), the identity of most of the lectican-negative cells (labeled by DAPI, Supplementary Figures 4E–H) is uncertain; they could be blood-derived, fibroblasts, or proliferated ependymal cells. A few lectican-positive neurons were present in the center of the glial injury reaction, based on fluorescent double labeling (white arrows, Supplementary Figure 5), but, like glial cells, most neurons were located outside the lesion core (arrowheads, Supplementary Figure 5D). Microglia/macrophages appeared in the TX site soon after injury (Figure 9). Additional ISH sections at 2 and 10 weeks post-TX showed that the microglia/macrophages also were labeled by lectican probes (black arrows, Figures 10G,H). Microglia/macrophages were concentrated in the center of the lesion soon after TX, but by 10 weeks, IB4-positive profiles had increased and spread away from the lesion site. Interestingly, many lectican-positive neurons also were reactive for IB4 (white arrows, Figure 10H). The identity of these neurons is unknown.

## Discussion

### Role of Lecticans in CNS Development

Anatomical plasticity declines in the CNS during postnatal development, with the appearance of PNNs, which enwrap neurons and proximal dendrites in the mature mammalian CNS. CSPGs are constituents of these PNNs (Carulli et al., 2006; Deepa et al., 2006; Kwok et al., 2011), and are upregulated after CNS injury, acting as major components of the glial scar that contribute to the failure of compensatory axon growth after SCI in mammals (Silver and Miller, 2004; Busch and Silver, 2007; Sharma et al., 2012). Expression of selected CSPG core proteins changes at the lesion site (Fitch and Silver, 1997; Lemons et al., 1999; Massey et al., 2008), and the removal of CSPGs by ChABC can counteract this growth inhibition (Bradbury et al., 2002; Huang et al., 2006; Cafferty et al., 2007), but no significant differences have been found with regard to the role of different lecticans in limiting post-injury axon growth (Warren et al., 2018b). The cellular sources of CSPGs, including lecticans, have been studied previously but are still not clear. Immunohistochemical experiments with lectins and antibodies recognizing CSPGs at both light and electron microscopic levels reveal reaction product interposed between and on neuronal surfaces, Therefore, the glial origin of this structure is widely accepted (Brauer et al., 1982, 1984; Bruckner et al., 1993). Immunohistochemical analysis of CSPG expression in cat visual cortex and primary rat cortical cultures reveals that different sets of neurons express different CSPGs, and that expression is regulated by neuronal activity (Lander et al., 1997). In addition, immunohistochemistry in primary rat cortical cultures demonstrates that neurons contribute to the extracellular matrix of the brain (Lander et al., 1998). However, because the antibodies used in these experiments were not well defined, the specific identities of the CSPGs are unclear. The composition and cellular origin of CSPGs that form PNNs were studied in the adult rat cerebellum with IHC and ISH (Carulli et al., 2006). Two lecticans, NCAN and ACAN, are produced only by neurons, BCAN mainly by glia and some neurons, and VCAN by NG2-positive cells and oligodendrocytes. The mRNA expression detected by ISH provided strong evidence that neurons express certain kinds of CSPGs, which form PNNs around their surface to stabilize synapses (Corvetti and Rossi, 2005; Bosiacki et al., 2019).

### Lecticans in the Lamprey Genome, and Their Role in CNS Development

Because lamprey is a primitive jawless vertebrate that diverged from jawed species early in vertebrate evolution, it has been included in the whole genome sequencing project (Smith et al., 2018a). In our search of the lamprey genome database, we found that lamprey retains numerous genes homologous to those of other species, including genes involved in the synthesis of myelin, even though the lamprey CNS is unmyelinated. Lamprey also has CSPGs and their RPTPs. Immunohistochemical study with and without ChABC digestion and lectin staining reveals that lamprey spinal cord neurons contain CSPG-rich PNNs (Hu et al., 2021) similar to those of mammals. Four lecticans are found in the lamprey genome, but phylogenetic and synteny analyses show that they lack clear one-to-one orthology with those of other species (Root et al., 2021). The present study showed that four lecticans are widely expressed in glia and neurons of the intact lamprey CNS, which is consistent with findings in mammals that lecticans are produced largely by two major cell groups in the CNS, neurons, and astrocytes (Siebert et al., 2014). Although the expression levels of lamprey lecticans showed individual differences, all four were high in embryos and young larvae, and declined before the adult stage. Lectican D dominated in embryos, whereas lecticans A and C became dominant at later stages of lamprey development. This suggests that coordinated expression of different lecticans may play an important role in axon growth and guidance during development, as in other vertebrate species, and that neurons, as well as glia, participate in the formation of PNNs.

### Lectican Involvement in “Scar” Formation After SCI

Our lab has long used lampreys to study CNS axon regeneration because of the heterogenous regenerating abilities of its RS neurons: some are good regenerators, and some are bad (Davis and McClellan, 1994; Jacobs et al., 1997). The bad regenerators also undergo very delayed retrograde cell death post-axotomy (Shifman et al., 2008). As others have reported (Bradbury et al., 2002; Massey et al., 2008), we found previously that levels of CSPGs in the lamprey glial scar increased to a peak at 2 weeks after SC-TX, and returned gradually to nearly normal levels by 10 weeks (Zhang et al., 2014a). Consistent with this, in the current study, the expression of lectican mRNAs demonstrated a peak at 1 week post-TX followed by a gradual decline. The large RS axons of lamprey retract during the first 2 weeks following SC-TX, then re-grow to and through the lesion scar after 4 weeks (Yin and Selzer, 1983). Immediately post-TX, blood elements, and fibroblasts fill the lesion gap. By 10 days, glial cells in the rostral and caudal stumps send processes into the lesion across (Lurie et al., 1994), followed by axons of small RS neurons and local interneurons (Zhang et al., 2020). By 4 weeks post-TX, as the lesion scar matures, the cellular elements decline and are replaced by an accessible longitudinal-glial fibrotic scar. After SC-TX glial processes in the scar were shown previously to change from a transverse to longitudinal orientation and serve as a bridge for regenerating axons to grow. The role of lecticans in determining the extent to which lamprey axons regenerate after SCI is not well understood. The increase in lectican levels during the time of glial scar formation and subsequent decrease indicates that CSPGs also participate in establishing the glial scar. However, lectican D mRNA expression levels remain high until 10 weeks, the longest time investigated. This expression pattern is analogous to the pattern of lectican expressions in the embryo. The molecular and cellular structures and functions of the scar tissues are highly dynamic after injury. In lamprey, after initial retraction, most lesioned axons regrow and reach or pass the lesion site by 4 weeks post-TX and continue to extend caudally, forming synaptic connections with some of their correct targets (Wood and Cohen, 1979; Mackler and Selzer, 1985, 1987; Cohen et al., 1988). This suggests that, as in early axon development, lectican D might play a particular role in the guidance of regenerating axons. How this would come about is not clear, since until now, selective interaction between specific lecticans and putative CSPG receptors has not been demonstrated.

### The Cellular Sources of Elevated CSPGs After SCI in Lampreys

Previous studies implicated reactive astrocytes as sources of proteoglycans *in vitro* (Fidler et al., 1999; Powell and Geller, 1999), and reactive astrocytes near the lesion have long been considered the major source of CSPGs after CNS injury (McKeon et al., 1991, 1995). Surprisingly, the removal of reactive astrocytes did not significantly attenuate the overall expression levels of CSPGs (Anderson et al., 2016). Many additional cell types contribute to glial scar formation and may modulate axonal growth and CNS repair after lesions, including fibroblasts, pericytes, oligodendrocyte progenitor cells, and inflammatory cells (Burda and Sofroniew, 2014; Li et al., 2021; Tran et al., 2022). Levels of CSPGs and their mRNA expressions were not significantly reduced at the site of an SCI lesion in astrocyte knockout mice, suggesting that both reactive astrocytes and non-astrocyte cells express inhibitory CSPGs (Anderson et al., 2016). In the current study on lamprey CNS, both glia and neurons expressed lecticans both before and after SCI, and if anything, neurons were more intensely labeled than glia. Most lectican-positive glia and neurons were located in the spinal cord adjacent to the site of injury, and only a few were in the lesion center. Instead, as shown previously, glial cells just rostral and caudal to the injury sites end longitudinally-oriented processes into the center of the lesion to form a bridge that supports the crossing of regenerating axons (Lurie and Selzer, 1991b; Lurie et al., 1994). The cells within the “scar” are mainly the ependymal and subependymal cells surrounding a reconstituted and widened central canal (Lurie et al., 1994). Although most of these cells are lightly labeled for lecticans, some are densely labeled. These expressed the neuronal marker Hu, and are probably CSF-contacting neurons. Thus far, they are the only neurons found to be proliferating after SCI in the lamprey (Zhang et al., 2014b).

After CNS injury, immune cells such as microglia/macrophages rapidly invade the lesion site, where they clear damaged tissue debris to help scar formation (Davalos et al., 2005; Dibaj et al., 2010). As in mammals, these microglia/macrophages bind to the plant lectin IB4 (Colton et al., 1992; Boscia et al., 2013). After SCI in lamprey, IB4-positive microglia/macrophages are significantly increased within the confines of the lesion site (Zhang et al., 2018a). The current study found that some IB4-positive microglia/macrophages also expressed lecticans, indicating that these immune cells are an additional source of the increased CSPGs after injury. Inflammation is a long-lasting response to SCI, and numerous IB4-positive profiles are present at 10 weeks in injured lamprey SC. Among them, some are small neurons. IB4-binding neurons have been reported in mammals (Stucky, 2013); many of them are sensory neurons that play a role in inflammatory pain. The role of IB4-positive neurons in lamprey is unknown. Thus, after SCI in the lamprey, glia, neurons, and immune cells all produce lecticans.

### Role of Lecticans in Axon Regeneration and Retrograde Neuronal Death After SCI

It is generally acknowledged that in mammals, increased CSPGs in the lesion site inhibit axon regrowth, and that reactive astrocytes are their main cellular source (McKeon et al., 1991; Pindzola et al., 1993; Silver and Miller, 2004; Alizadeh et al., 2021). However, it has also been reported that ablation of reactive astrocytes suppresses spontaneous regrowth of axons through the scar and increases retraction of corticospinal tract axons, supporting the idea that reactive astrocytes around the lesion are important for sustaining tissue integrity and increasing regenerative ability after SCI (Anderson et al., 2016). Interpretation of those results has been complicated by the cellular complexity of the injury reaction, which extends beyond reactive astrocytosis and includes invasion of the injured area by inflammatory cells and other potentially growth-inhibiting cell types that comprise the scar tissue (Silver, 2016). Nevertheless, in recent years, it has been widely accepted that the “glial scar” may play a dual role in SCI recovery, even if the precise roles of the astrocytes themselves are not clear (Yang et al., 2020). Because of the long-life cycle of the lamprey, we have not used a transgenic or germ cell genetic strategy to address this issue. However, enzymatic digestion of CSPGs by local application of ChABC to the injury site early after SC-TX enhanced axonal regeneration and reduced retrograde death of RS neurons (Hu et al., 2021). Removing CSPGs at the site of injury also prevented early axon retraction in the rostral stump. This observation was based on the large RS axons, which belong to the large, identified RS neurons. These generally are worse regenerators than the smaller RS neurons, and they also usually undergo a very delayed retrograde cell death (Shifman et al., 2008; Zhang et al., 2018b). Axon retraction is greater in large caliber than small caliber RS axons, many of which already have regenerated into or past the lesion site by 2 weeks post-TX (Zhang et al., 2020). Moreover, RS neurons with poor regenerating probability selectively express the RPTP receptors for CSPGs (Zhang et al., 2014a). The present findings in lamprey are consistent with the idea that different CSPGs might either repel or support axon growth after SCI. Early after SCI, CSPGs might promote retraction and inhibit the growth of giant axons that express CSPG receptors and participate in glial process bridge formation. Upregulation of lectican D was particularly strong and persistent, at least to 10 weeks post-TX, when many smaller propriospinal and reticulospinal axons are already regenerating into the scar (Zhang et al., 2020). This suggests that post-SCI, some CSPGs, particularly lectican D, might actually promote axon growth or guidance. Whether this is true has not been tested *in vivo*. Although lamprey lecticans are not orthologous with those of gnathostome vertebrate species, lectican D is distinctly homologous with NCAN, which in the rat, was upregulated in both knife and contusion models of SCI. After contusion, NCAN levels persisted both at the injury site and remotely for at least 28 days post-injury, whereas aggrecan and brevican were reduced at the injury site after contusion (Andrews et al., 2012).

Work in lamprey suggests that axon regeneration after SCI does not involve the formation of conventional growth cones, which characterize developing axons during embryogenesis and axon outgrowth *in vitro*. Instead, the leading edge of regenerating RS axons are simple in shape, lack filopodia, have little F-actin, and are filled with densely packed, highly phosphorylated neurofilaments (Lurie et al., 1994; Pijak et al., 1996; Hall et al., 1997; Zhang et al., 2005; Jin et al., 2009). Nevertheless, regeneration of lamprey RS axons shares several features in common with mammalian CNS axon growth during development, including inhibition by CSPGs (Sharma et al., 2012; Hu et al., 2021), RhoA signaling mediating retrograde cell death and inhibition of axon growth post-axotomy (Dubreuil et al., 2003; Fujita and Yamashita, 2014; Hu et al., 2017; Zhang et al., 2018a), and promotion of axon growth by cAMP (Cai et al., 2001; Jin et al., 2009). The selective upregulation of lectican D during embryogenesis and again after spinal cord injury in the lamprey is another possible point of convergence between development and regeneration.

## Data Availability Statement

Any original data not presented in the article and Supplementary Material will be made available upon request directed to the corresponding author.

## Ethics Statement

The animal study was reviewed and approved by Temple University Institutional Animal Care and Use Committee.

## Author Contributions

GZ designed and conducted the experiments, acquired the images, carried out the data analysis, and drafted the manuscript. L-QJ conducted phylogenetic analysis of lectican genes. WR cloned a fragment of lamprey lectican and contributed to ISH. JH helped prepare the manuscript. ZR and DM designed templates for synthesizing probes of Lec A, B, C, and D. MS contributed to the overall design of the study and to the design of some specific experiments and helped write the manuscript. All authors contributed to the article and approved the submitted version.

## Conflict of Interest

The authors declare that the research was conducted in the absence of any commercial or financial relationships that could be construed as a potential conflict of interest.

## Publisher’s Note

All claims expressed in this article are solely those of the authors and do not necessarily represent those of their affiliated organizations, or those of the publisher, the editors and the reviewers. Any product that may be evaluated in this article, or claim that may be made by its manufacturer, is not guaranteed or endorsed by the publisher.
