# Supplementary material for: The Composition and Cellular Sources of CSPGs in the Glial Scar After Spinal Cord Injury in the Lamprey
Source: Front Mol Neurosci. 2022 Jun 27;15:918871. doi: 10.3389/fnmol.2022.918871 (PMC9271930; doi:10.3389/fnmol.2022.918871)
Supplement: Supplementary file 1 [file Table_1.docx]

**Supplementary Table 1. NCBI accession numbers used for the phylogenetic analysis**

| Name | Access No. | Length (aa) | notes |
| --- | --- | --- | --- |
| ACAN *(Bos taurus)* | XP_024837358 | 2366 | core protein |
| ACAN (*Oryctolagus cuniculus*) | XP_008249943 | 2167 | core protein |
| ACAN (*Mus musculus*) | NP_001348429 | 2170 | core protein |
| ACAN (*Homo sapiens*) | NP_037359 | 2530 | isoform 2 |
| ACAN (*Rattus norvegicus*) | EDM08568 | 2162 | Isoform CRA_a |
| VCAN *(Bos taurus)* | NP_851378 | 3381 | Core protein |
| VCAN (*Oryctolagus cuniculus*) | XP_017200054 | 3424 | Isoform X1 |
| VCAN (*Mus musculus*) | NP_001074718 | 3354 | Isoform 1 precursor |
| VCAN (*Homo sapiens*) | NP_004376 | 3396 | Isoform 1 precursor |
| VCAN (*Rattus norvegicus*) | NP_001164029 | 3357 | Isoform 1 precursor |
| NCAN *(Bos taurus)* | XP_015327358 | 1385 | Isoform X1 |
| NCAN (*Mus musculus*) | NP_031815 | 1268 | precursor |
| NCAN (*Homo sapiens*) | NP_004377 | 1321 |  |
| NCAN (*Oryctolagus cuniculus*) | XP_008251641 | 1435 |  |
| NCAN (*Rattus norvegicus*) | NP_113841 | 1263 | protein precursor |
| BCAN *(Bos taurus)* | XP_005203663 | 912 | Protein isoform X1 |
| BCAN (*Oryctolagus cuniculus)* | XP_008262524 | 912 | core protein |
| BCAN (*Mus musculus*) | XP_030108256 | 900 | isoform X2 |
| BCAN (*Homo sapiens*) | XP_016857536 | 956 | Isoform X1 |
| BCAN (*Rattus norvegicus*) | NP_001028837 | 883 | Isoform 1 precursor |
| VCAN x1 (*Petromyzon marinus*) | XP_032805506 | 2834 | VCAN-core protein like, X1 |
| ACAN x1 (*Petromyzon marinus*) | XP_032829584 | 3331 | ACAN-core protein like, X1 |
| VCAN (*Petromyzon marinus*) | XP_032824522 | 3706 | VCAN-core protein like |
| NCAN x1 (*Petromyzon marinus*) | XP_032831863 | 1712 | NCAN-core protein like, X1 |
| PTPRZ1 (Homo sapiens) | NP 002842 | 2315 | Isoform 1 precursor |
