# Supplementary figures and images for: The Composition and Cellular Sources of CSPGs in the Glial Scar After Spinal Cord Injury in the Lamprey
Source: Front Mol Neurosci. 2022 Jun 27;15:918871. doi: 10.3389/fnmol.2022.918871 (PMC9271930; doi:10.3389/fnmol.2022.918871)

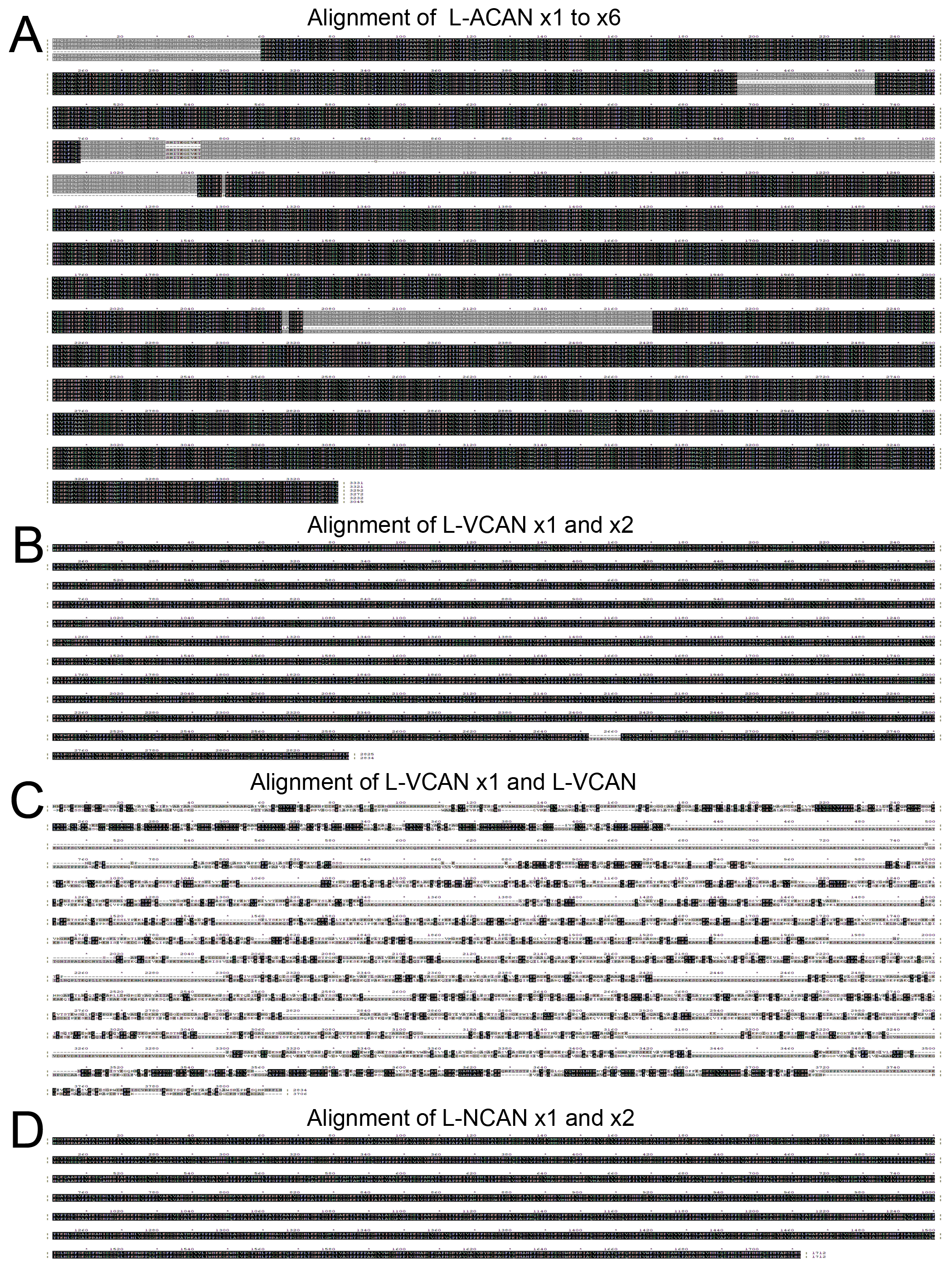

Supplement: Supplementary file 2 [file Image_1.TIF]

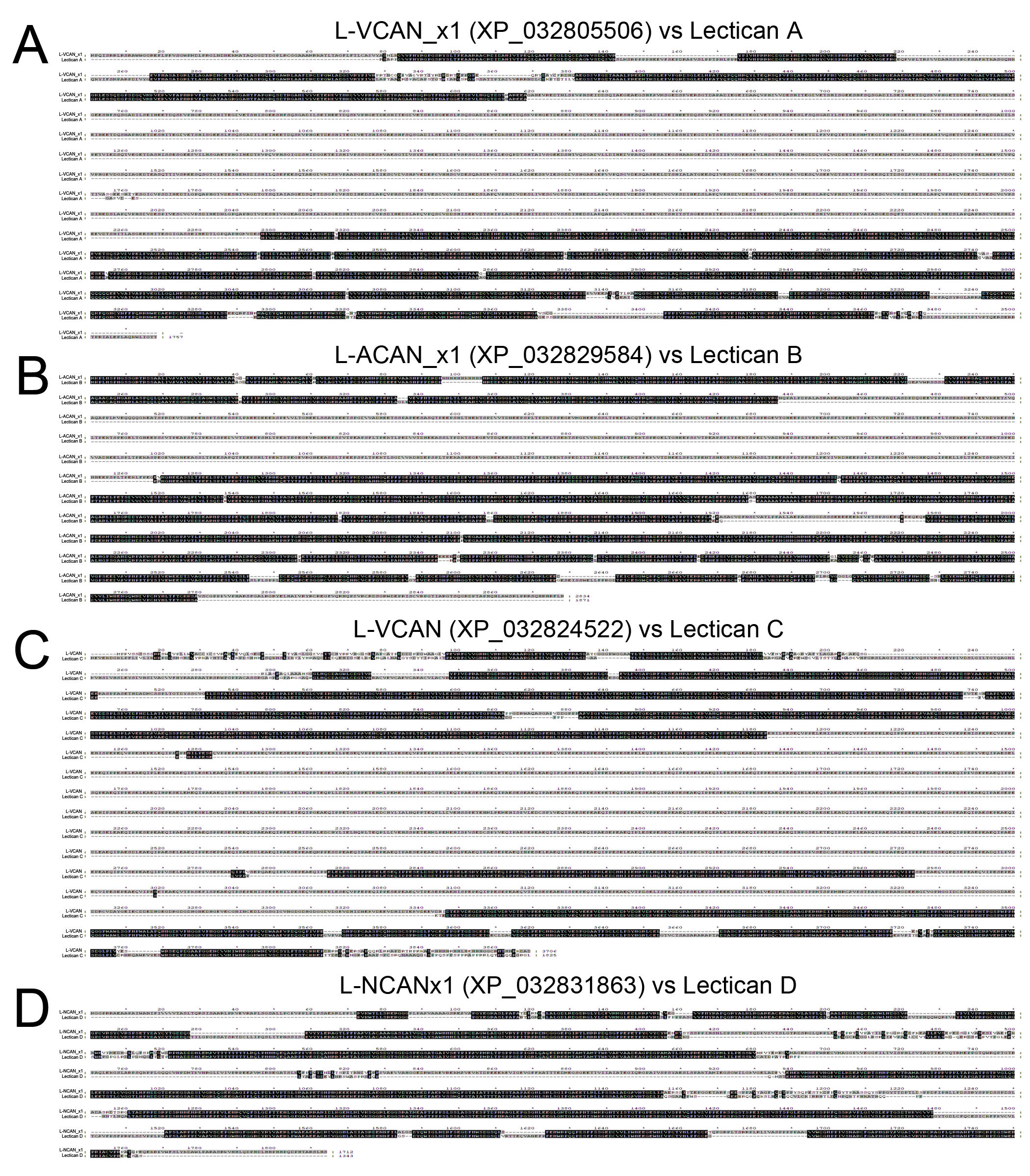

Supplement: Supplementary file 3 [file Image_2.TIF]

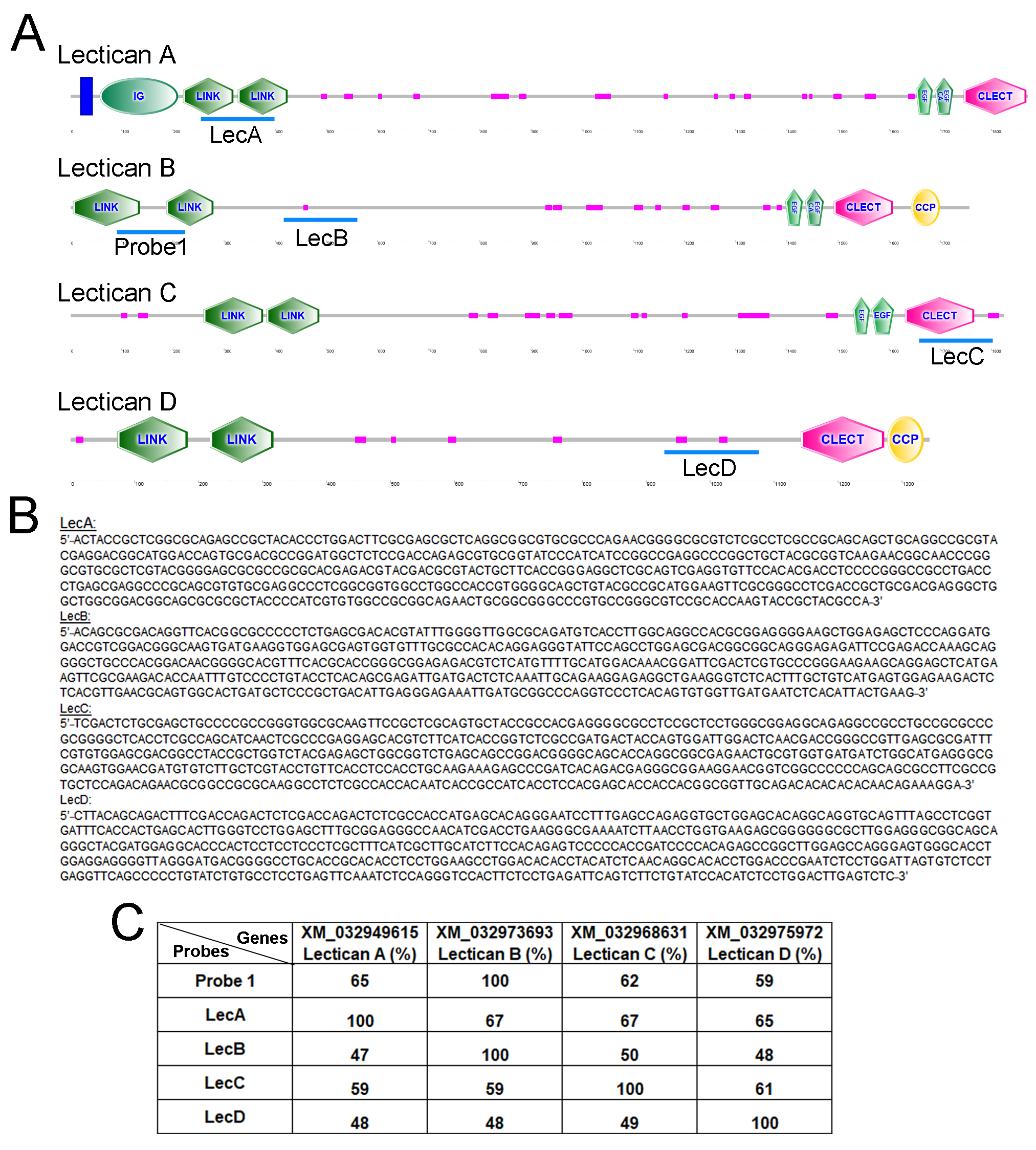

Supplement: Supplementary file 4 [file Image_3.TIF]

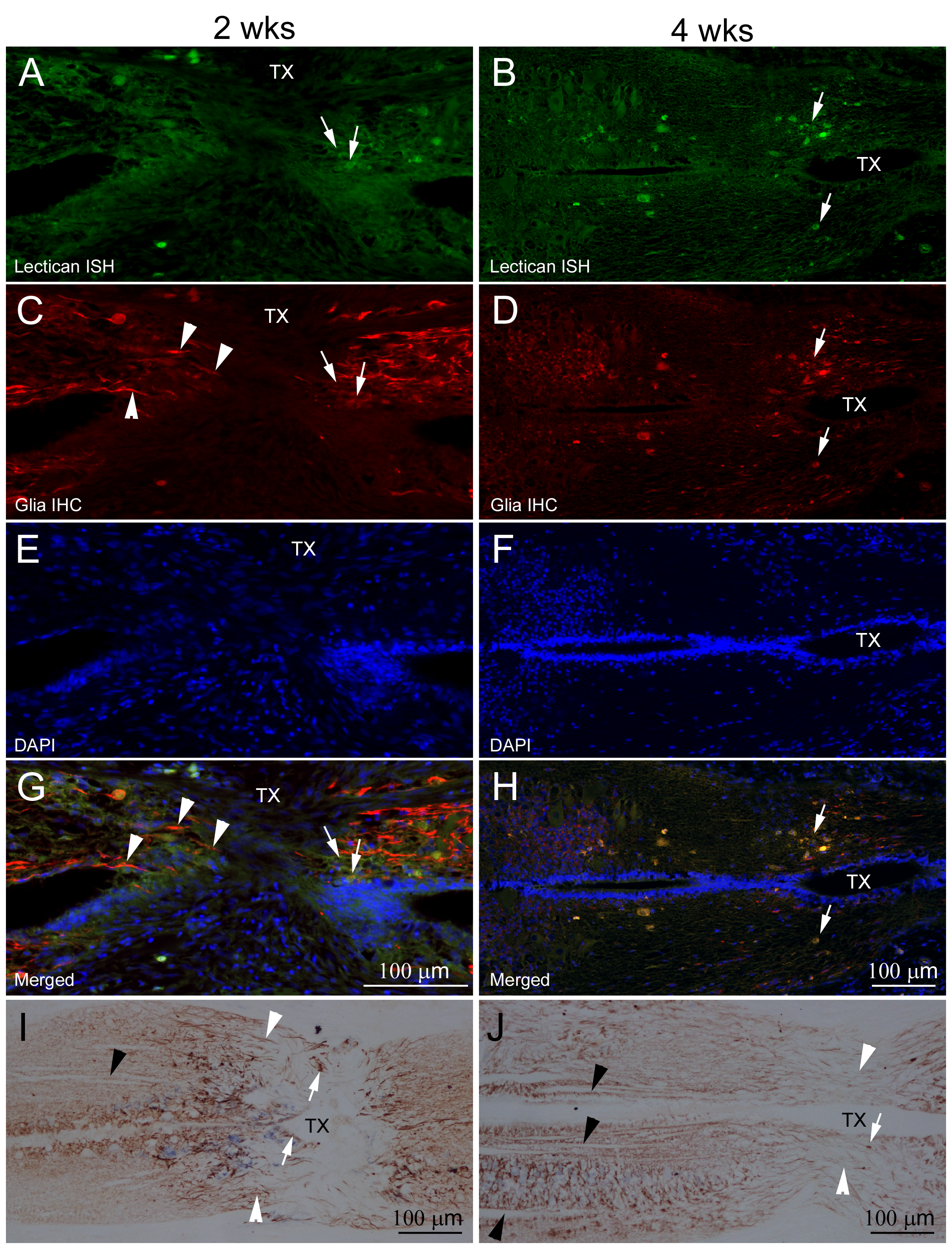

Supplement: Supplementary file 5 [file Image_4.TIF]

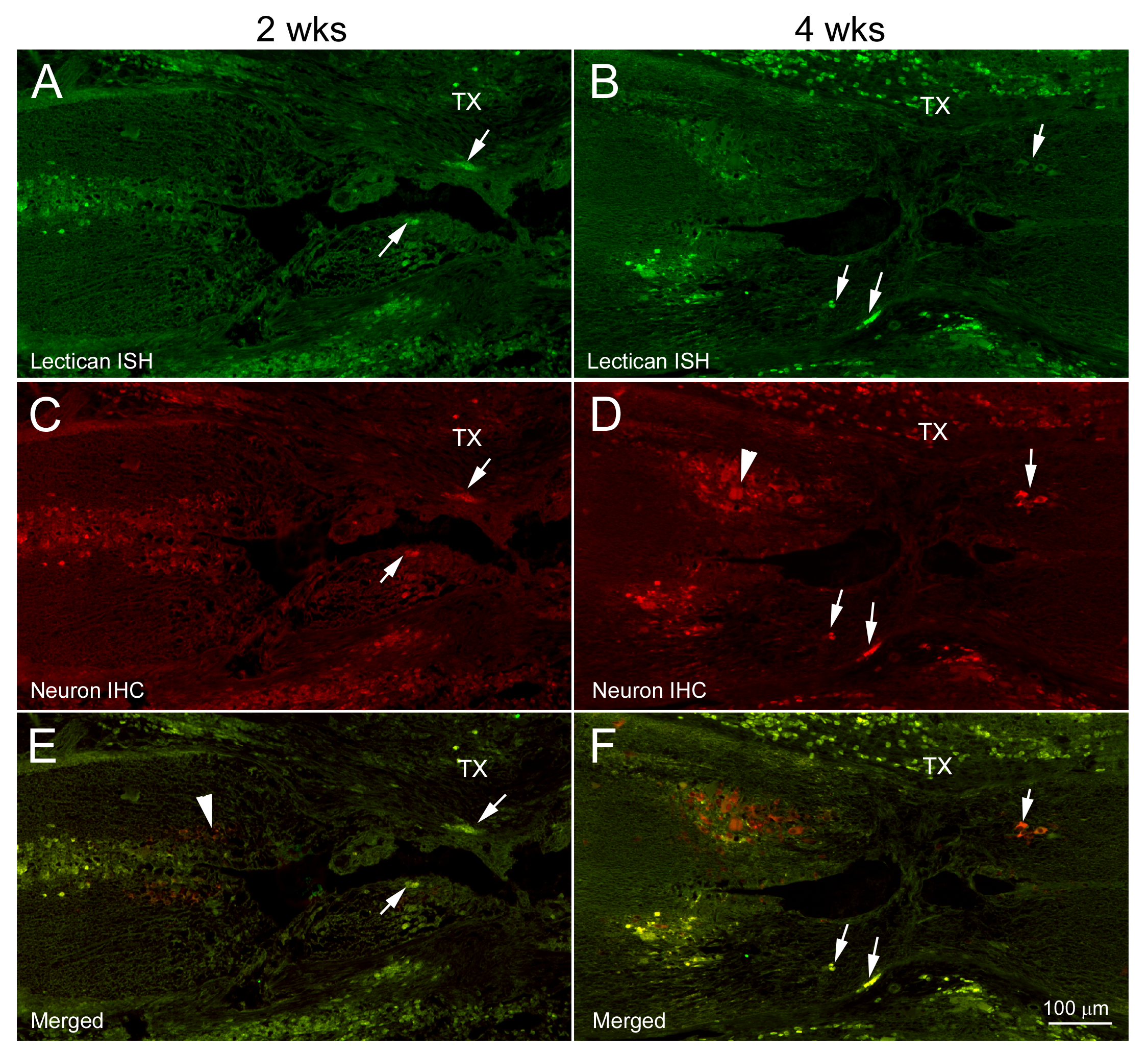

Supplement: Supplementary file 6 [file Image_5.TIF]
